# Improving follow-up care for people after minor stroke using early personalised care: A protocol for a randomised, mixed-methods, feasibility study.

**DOI:** 10.3310/nihropenres.13649.3

**Published:** 2025-02-26

**Authors:** Jennifer Crow, Hilary Watt, Mary Wells, Paresh Malhotra

**Affiliations:** 1 Department of Occupational Therapy and Physiotherapy, Imperial College Healthcare NHS Trust, London, UK; 2 Department of Brain Sciences, Imperial College London, London, England, UK; 3 Department of Primary Care and Public Health, Imperial College London, London, England, UK; 4 Department of Surgery and Cancer, Imperial College London, London, England, UK; 5 Nursing Directorate, Imperial College Healthcare NHS Trust, London, UK

**Keywords:** Minor stroke, complex intervention, feasibility study, secondary prevention, follow-up care, hidden impairments.

## Abstract

**Background:**

Of the 150 000 people per year in the UK who have strokes, third to half will experience a so-called ‘minor stroke’. Although appearing benign these strokes put a person at increased risk of further strokes and survivors are usually considered ‘too good’ for referral onto community stroke services. When back at home the hidden effects of stroke like fatigue and changes in mood and cognition become apparent and impact return to work, relationships and everyday activities. Alongside this, managing the risk of recurrence, highest early after an initial stroke, is a priority. People with stroke report feeling abandoned after discharge with unmet information and support needs.

**Methods:**

To address this issue, we reviewed the literature, met with people with stroke and other stakeholders to develop an early, personalised follow-up programme of care for those who currently only receive routine medical follow-up appointments. The design of this complex intervention is informed by the NIHR/MRC framework for complex intervention development and the framework of action described by O’Cathain and colleagues. The intervention is underpinned by self-determination theory. We will be conducting a randomised, mixed methods, single-centre feasibility study to explore the acceptability and feasibility of the intervention. Sixty participants will be recruited from a Hyperacute Stroke Unit and Rapid Assessment Clinic and randomised to the intervention or control group. The intervention group will receive personalised follow-up appointments at two- and six-weeks post-discharge. All participants will have outcome measures taken at baseline and twelve-weeks post-stroke. Patient reported outcomes will be reviewed to assess their suitability for a later definitive trial. Qualitative interviews will be conducted to gain a deeper understanding of life after stroke from those who did and did not receive the intervention.

**Conclusions:**

Study findings will be used to further refine the intervention, methods and outcome measurements used. These refinements will inform a future multicentre randomised controlled trial.

## Plain Language Summary

**What is the background to this research?:**

The term minor stroke refers to a stroke that has caused damage to a person’s brain that is visible on a brain scan, but the person has no obvious physical, thinking, or speaking problems. The stroke team think the person has made a good recovery and subtle stroke effects go unnoticed. The hospital stay is short and no onward therapy referrals are made.

When back at home people realise they need more information about their stroke and how to reduce the risk of another stroke. As the person starts resuming their busy lives they notice the impact of fatigue, cognitive and mood changes. Getting stroke specific support at home is hard and people say they feel abandoned.

**What is the intervention that is being suggested?:**

Together with people who have experienced minor stroke we developed a follow-up programme of care to address this problem. A stroke therapist will conduct personalised follow-up appointments at two- and six-weeks after discharge. The appointments will focus on addressing unmet information needs and providing support and guidance to adjust to life after stroke. Referrals to community services will be made if needed.

**How will the research be done?:**

We will recruit participants from a hyperacute stroke unit and rapid assessment clinic. They will be randomised to receive the intervention or usual care. This feasibility study will tell us if people find the study processes, follow-up appointments and questionnaires acceptable. Both groups will complete questionnaires at baseline and 12-weeks and will be invited to take part in qualitative interviews.

The learning from this feasibility study will let us know what changes and improvements need to be made before conducting a larger study to see if these personalised, follow-up appointments will improve the lives of people after a stroke.

## Introduction

### Background and rationale

***The size of the problem***

Stroke is the leading cause of disability in the UK and there are 1.3 million people living with the after-effects of stroke ^1^. Of the 150 000 strokes occurring per year, a third to a half of these are minor strokes ^2, 3^. The incidence and prevalence of stroke are increasing ^4^ and the economic burden of stroke on society, health, social care, and the person and their family is significant ^5^. A large population-based study in the United Kingdom showed a decline in stroke mortality rates ^6^. However, there has been an increase in the event rates in the 35–54 age group ^6^. The total annual societal cost of stroke in the UK is £26 billion; the largest portion of these costs, £20.6 billion, relates to the provision of ongoing care ^5^. 

***The importance of the problem***

Large-scale policy initiatives, such as the NHS Long Term Plan ^7^ and the Stroke Plan for Europe ^8^ have recognised the importance of improving stroke services. They are calling for a focus on improving life after stroke by improving how services work together and empowering people with stroke to manage their risk of further stroke and optimise their quality of life. Stroke shares many risk factors with cardiovascular disease (CVD), one of the largest contributors to health inequality ^9^. One-fifth of the life expectancy gap between the most- and least-deprived communities is attributable to cardiovascular disease. Stroke is more common in people from South Asian and Black communities and they have the highest risk of CVD ^9^. Stroke and cardiovascular disease can often be prevented by leading a healthy lifestyle; however, this requires communities that are supported, informed, and engaged. We also know that evidence-based treatments to reduce recurrence of stroke exist and yet evidence from a large South London Stroke Registry study has shown that the incidence of secondary stroke has not reduced since 2005 ^10^. These data indicate that the implementation of secondary prevention programmes has not yet been optimised.

The James Lind Alliance research priority setting partnership ensures that the voices of people with stroke carry equal weight to those of clinicians and researchers. Their stroke priority setting partnership report identified priority research areas that align with this research ^11^:

- What can stroke survivors do to reduce the risk of having another stroke?
- What are the best interventions to promote recovery and prevent further stroke in people who have had strokes?
- How can we recognise, assess, treat, and support people who experience hidden impairments (psychological problems, cognitive and communication changes, and fatigue) after a stroke?

***What is a minor stroke?***

There is no universally agreed definition for minor stroke ^12, 13^ and the terminology used, such as minor, mild, mini, and non-disabling, is confusing. In this feasibility study, we will use the tissue-based definition ^14^ for minor stroke (a stroke visible on imaging). *The definition we use for TIA is the presence of transient neurological symptoms which appear to have fully resolved within 24 hours and the absence of tissue damage on brain imaging. The National Institute for Health Stroke Scale (NIHSS) score is an impairment scale which is widely used in clinical practice and research to define minor, moderate or severe stroke (see Table 1) ^15^. The most commonly used cut off points for minor stroke in research studies are NIHSS ≤ 3 or NIHSS ≤ 5 ^16^. We will be using NIHSS ≤ 5 as our cut off point in this study.*

**Table 1.** Modified Rankin scale.

Level	Description
0	No Symptoms
1	No significant disability, despite symptoms; able to perform all usual duties and activities
2	Slight disability: unable to perform all previous activities but able to look after own affairs without assistance.
3	Moderate disability: requires some help, but able to walk without assistance.
4	Moderate severe disability: unable to attend to own bodily needs without assistance and unable to walk unassisted.
5	Severe disability: requires constant nursing care and attention, bedridden, incontinent.

*In our service the medical management of those with both minor stroke and TIA focuses on the identification and management of vascular risk factors. Both people with a diagnosis of TIA or minor stroke will have a therapy assessment on the HASU. Only those with a minor stroke are referred for a six-month stroke review. Community stroke therapy teams that subscribe to the time-based definition for TIA will usually not accept referrals for people with a diagnosis of TIA*.

Hyperacute treatments such as thrombolysis and thrombectomy improve survival post-stroke, and increasing numbers of people are discharged home with minimal physical disability. This scenario requires further attention from healthcare services and society. People with stroke say that a life saved is a life worth living, and services need to improve and change the focus from surviving to thriving ^17^.

***What is a complex intervention?***

Many interventions used in healthcare are considered to be complex interventions, whereby the intervention consists of a number of different interacting components, each targeting different areas of behavioral change or aspects of health and wellbeing. Complex interventions allow a degree of flexibility as they are applied in different contexts or different systems to a wide range of individuals with varying levels of health literacy and from a diverse range of communities ^18^.

***What happens in current practice?***

People with minor stroke typically have a very short length of stay in hospital or are increasingly being managed in outpatient clinics and emergency departments ^19^. The clinical team focuses on ruling out obvious physical, cognitive, and communication difficulties. The assessment tools used are generally insufficiently sensitive to pick-up high-level impairments ^20, 21^. People consistently report that they receive insufficient personalised information prior to discharge ^22^. The discharge summary is written with the general practitioner in mind and is often difficult for laypeople to understand. On discharge home, the person with stroke describes feeling abandoned and lost ^22, 23^. Given the lack of information provision and the inaccessible nature of discharge summaries to lay persons, it is not surprising that up to a third of people with stroke and TIA will have discontinued their medication within a year of discharge ^24^.

Current medical follow-up appointments are brief and focus specifically on medical issues and outstanding medical investigations. Ideally, these should occur 6 weeks post-discharge, but in practice, the wait for these appointments is often longer. These appointments rarely have multidisciplinary input; therefore, other issues relating to hidden effects, adjustment to life after stroke, and secondary prevention are seldom addressed. National Clinical Guidelines for Stroke (2023) recommend that all people after stroke should receive a holistic and structured 6-month review ^25^.

We conducted a scoping review to identify follow-up programmes, pathways, and services that have been developed for people after minor stroke ^26^. Twenty-five studies were included in the analysis. Interventions often focused on education, secondary prevention, and exercise after stroke, whereas the management of hidden impairments and adjustment to life after stroke were less likely to be addressed. These complex interventions were often poorly described, and descriptions of family involvement, liaison between primary and secondary care, and underpinning psychological or behavioural theories were lacking. The review revealed that it is still unknown how best to organise services to offer personalised timely support and education to people in the community. There were two RCTs included in the scoping review, (STROKE-CARD ^27^ and ‘Take Charge’ ^28^) that were able to demonstrate improvements in health-related quality of life at 12 months after the stroke. STROKE-CARD involved personalised, comprehensive multidisciplinary follow-up at 3 months post stroke and the Take Charge follow-up intervention was underpinned by self-determination theory and involved either one or two follow-up appointments. Take Charge’s person centred intervention focused on supporting a person to take charge of their recovery through supporting self-empowerment. The key elements from these two studies; multidisciplinary focus, personalised care and self-determination theory are included in the intervention described in this protocol. We believe that this may increase the likelihood of being able to show meaningful improvements in this diverse patient population.

The objective of this feasibility study is to determine if an early, personalised, programme of care involving holistic follow-up of people with minor stroke discharged from the hyperacute stroke unit (HASU) and rapid assessment clinic (RAC) is acceptable and can feasibly be delivered within the NHS.

It will address the following uncertainties:

- Recruitment rate (percentage of eligible people agreeing to take part).
- Treatment adherence rate (percentage of people attending both follow-up appointments and engaging in agreed actions).
- Participant retention rate (percentage of those in the intervention and control groups that complete the final outcome measurement).
- Acceptability and accessibility of outcome measures.
- Ability to gather self-report data to complete an economic evaluation.
- Acceptability and perceived benefit of the follow-up intervention.

If the feasibility objectives meet the predefined progression decision criteria and the intervention and outcome measures are accessible and acceptable to those with minor stroke, then the next stage will be to deliver a definitive multicenter trial to determine the efficacy of this complex intervention.

## Methods

### Patient and Public Involvement

A patient and public involvement group with experience of minor stroke was instrumental in the study conception and design. Three online group meetings with six attendees and the lead investigator took place to plan and design the study. A further two face-to-face meetings with people from underserved communities who did not wish to attend online group meetings were also held. Their input highlighted the challenges of online meetings, reading study documentation, and completing outcome measures on a mobile phone for those who do not have access to laptops or desktops. In the online and face-to-face meetings, the importance of the research idea was explored, the research question was agreed upon, the intervention was developed and refined, and this included participants’ input shaping the frequency and content of the follow-up appointments. An underpinning theory was presented, and its relevance and understandability were critiqued by the group. Outcome measures originally considered were excluded based on group feedback, and the final measures were agreed upon.

To improve the inclusivity of this research, perspectives and feedback from a diverse range of people were obtained. This included presenting the planned research to a group of ethnic minority women from an underserved community. Through this group, an improved understanding of the barriers and concerns of this community regarding follow-up care was developed, and recruitment processes were adapted accordingly. Our team was exposed to different health belief systems and attitudes towards disease and recovery. In addition, a group member reviewed the information sheet and the consent form. Based on her feedback, an accessible supplementary information sheet was developed, and improvements were made in the consent form.

Engagement with acute and community-based stroke clinicians in localities beyond our own confirmed that the lack of structured follow-up care for people after minor stroke also exists in other services. We learned about clinical services that are attempting to deliver different models of follow-up care to complement the narrow focus of stroke medical follow-up appointments. Addressing secondary prevention, adjustment to life after stroke, and assessment of ongoing therapy needs requires additional multidisciplinary staff in the clinic. Services described difficulties in obtaining substantive funding to enable stroke follow-up clinics to provide more holistic and personalised care. We are also engaged in an international collaboration with other researchers in this field. We are conducting a systematic review to collate the latest evidence on the lasting impairments experienced after minor stroke and TIA ^29^.

### Trial design

**Figure 1.**
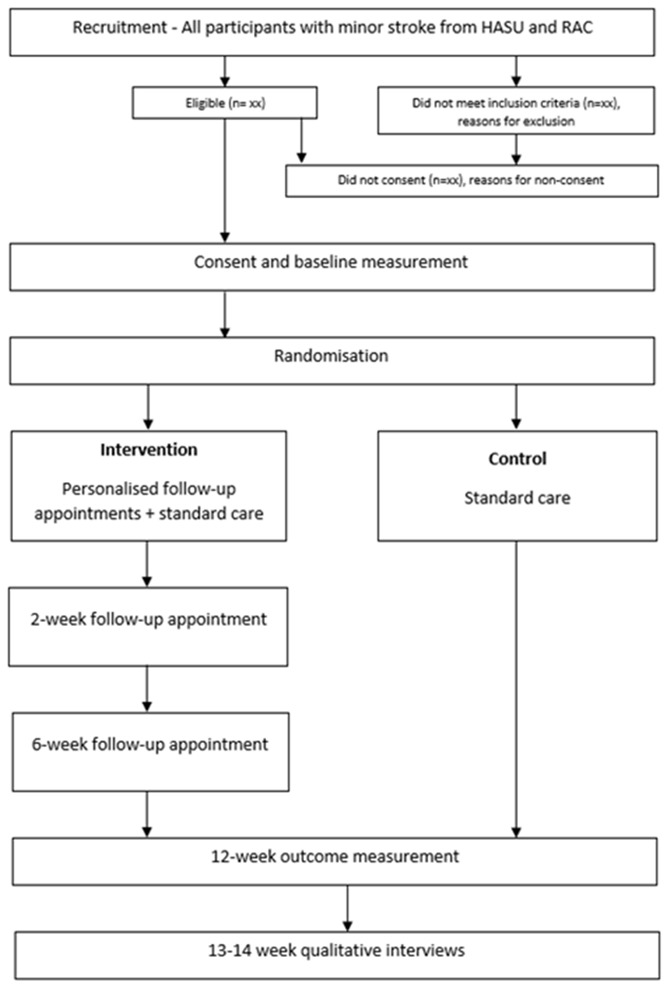
Consort diagram.

The current trial is a two-armed, parallel group, randomised, single-center, feasibility study with embedded qualitative interviews. Participants and the outcome assessor, a specialist occupational therapist (OT), will not be blinded to the intervention. The study flowchart is shown in the Consort Diagram Figure 1.

This protocol has been reported in accordance with SPIRIT guidelines ^30^. The template for the intervention description and replication (TIDieR) checklist was used to report the intervention details ^31^. These documents can be found in the Open Science Framework Repository. https://osf.io/gnw57/

### Setting

Recruitment: Participants with a NIHSS score of ≤5 at 2 hours post admission and meeting the other inclusion criteria will be recruited from the HASU and RAC. People attending the RAC are referred to the clinic via their GP or may have been seen in the emergency department and discharged home but with follow-up investigations to be completed in the clinic. Both the HASU and RAC are located in a high-density, ethnically diverse, urban environment.

Outcome measurement: Baseline measures for both groups will be conducted by the OT in the hospital where possible, but if the person is discharged quickly or seen in the RAC, then they will be conducted at the persons home or in the clinic. The final outcome measurement location will be agreed upon by the participant and could be in their home or in the hospital.

Intervention: Participants will determine their preferred location for follow-up appointments. They will be offered in-person appointments in the outpatient clinic at the hospital, appointments over the telephone or video call, or in their home or neutral venue in the community.

This personalised approach is based on PPI input into the study design and aims to increase the inclusivity of the study. Online appointments introduce digital exclusion and favour younger participants. On the other hand, having to travel to the hospital may be a deterrent for older participants or those who are back at work.

### Trial registration

ClinicalTrials.gov - NCT05897905 (22/05/2023)

Accessed at: https://classic.clinicaltrials.gov/ct2/show/NCT05897905

### Protocol version

Protocol V4 (31/03/2023), approval date 21/07/2023.

Substantial amendment - SA01 – Protocol V5 (26.10.2023), approval date 06/02/2024.

### Eligibility criteria

***Inclusion criteria***

- Adult ≥18
- Clinical or radiological diagnosis of minor stroke from a stroke consultant.
- Admitted to HASU or seen in RAC.
- Pre-admission modified Rankin Score (mRS) of 0–3 (see table below), with no formal package of care prior to stroke. Table 1. Modified Rankin Scale scores ^32^
- NIHSS ≤ 5 at 2 hours post admission. Table 2. Stroke Severity – National Institute of Health Stroke Scale Score
- Within one week of receiving stroke diagnosis.
- Discharge mRS of 0–3.
- No onward referral to community therapy team or for package of care.
- Has capacity to consent.

**Table 2.** Stroke Severity – National Institute of Health Stroke Scale Score.

NIHSS Score	Stroke Severity
0	No Stroke symptoms
0–4	Minor Stroke
5–15	Moderate Stroke
16–20	Moderate to Severe Stroke
21–42	Severe stroke

***Family member inclusion criteria for qualitative interview (SA-01)***

- Family members or partners who participated in and/or supported the researcher with the recruitment process, intervention delivery, and outcome measurement.

***Exclusion criteria***

- Any serious comorbidities that would impact a person's ability to participate in follow-up appointments and outcome measurement.
- Insufficient English to engage in intervention and outcome measures, and no family or friends to support with this.
- Not resident in England.
- Pregnant women.
- Enrolled in another interventional trial that does not allow co-enrolment or observational studies where the burden of assessment may be too high.

### Participant identification, screening and recruitment

The researcher will review the electronic records of all new admissions to the HASU. If the patient meets the study criteria, eligibility is confirmed by the assessing team. This process is followed for weekend admissions. If the researcher is unable to attend the ward in person prior to discharge, then consent for contacting the patient post-discharge will be obtained and documented. In the RAC, potential study participants will be identified by stroke clinical nurse specialists, and consent will be obtained for the researcher to contact the person following discharge. Consent for participation in the study will be completed by the researcher either on the ward or in the clinic or at the participant’s home.

Data will be collected to capture the baseline and clinical characteristics of those approached for study involvement as well as their ethnicity, and level of education ( Table 3).

**Table 3.** Participant data collection domains.

Pre-Stroke Information	Admission Information	Discharge Information
mRS Past medical history Clinical Frailty Scale (CFS) Employment status PHQ2 EDI information: - Ethnicity - Age - Education level	NIHSS* Hyperacute treatments • Thrombolysis • Thrombectomy • Both • Neither	NIHSS mRS MoCA Length of hospital stay

Table 3 key: mRS-modified Rankin Scale, PHQ2-patient health questionnaire 2, EDI-ethnicity, diversity and inclusion, NIHSS-National Institute of Health Stroke Scale, MoCA-Montreal cognitive assessment.

Strategies to promote recruitment: Therapy staff within the stroke service are rotational; therefore, regular study updates will be given to the therapy team by attending their fortnightly team meetings. A template of what to write in the patient record when approaching a potential study participant will be provided, and the study inclusion and exclusion criteria will be clearly displayed on the therapy information board on the ward.

The recruitment target is five participants per month from the HASU. This target is based on pilot data; people with minor stroke were recruited from the HASU over a three-month period to a two-week follow-up study involving online cognitive testing ^33^. No preliminary data gathering was conducted at the RAC, but time was spent in the clinic to understand what a clinic appointment involves for the patient. A smaller proportion of people who attend this clinic will receive a diagnosis of stroke; therefore, the recruitment target is set at two participants per month.

### Randomisation

Participants will be randomised to the intervention or control group by simple randomisation using a 1:1 allocation ratio and opaque sealed envelopes to ensure allocation concealment. Randomisation will be stratified by recruitment location to ensure treatment assignment is balanced between the two groups within each location.

### Sample size

Progression criteria related to feasibility outcomes were identified. We plan to recruit 60 participants (30 in the control group and 30 in the intervention group) in line with sample sizes for similar feasibility studies ^34^. Feasibility studies need to be much smaller than full-efficacy trials to be considered worthwhile. The feasibility outcome “participant retention” is the percentage of 60 participants who complete outcome measurements at 12 weeks. Using a binomial probability confidence interval (CI) calculator with a sample size of 60, the 95% CI on proportions will have widths no greater than 37% (based on the 50% proportion having a 95% CI from 37% to 63%. Proportions further from 50% would have narrower CIs; for example, 90% has a 95% CI from 79–96%, so this CI has a width of 17%. Free Binomial Probability Confidence Interval Calculator - Free Statistics Calculators (danielsoper.com)

The width of these CIs provides a reasonable balance between the cost and practicality of patient recruitment. The feasibility outcome “recruitment uptake” would have narrower CIs (since 60 is the numerator with a larger denominator). There is no relevant power calculation because the main outcomes are proportions rather than anything that can be assessed using hypothesis testing.

The progression criteria targets are described in Table 4. They provide a guide through the use of a predefined traffic light system of red (stop), amber (amend, modifications needed), and green (proceed), as to whether the study could proceed to a future definitive trial. The targets may seem ambitious, but due to the limited number of appointments and personalised nature of the intervention, where participants do not need to attend appointments at the hospital, we believe these are achievable. We have carried out extensive work with diverse community groups that has improved our understanding of culturally appropriate ways to approach patients from different communities. Further, this is also a single-centre study, with the lead researcher having established working relationships with the other members of the stroke team.

**Table 4.** Progression decision criteria.

FEASIBILITY OBJECTIVES	MEASURES USED	DENOMINATOR FOR PERCENTAGE	GREEN Proceed to full scale trial	AMBER Amendments needed	RED Definitive trial unlikely to be feasible
1. Recruitment uptake	% of eligible people agreeing to take part	Over 60 (numerator is 60)	≥90%	60–89%	<60%
2. Treatment adherence	% of participants that attend both follow-up appointments	30 (patients recruited into treatment arm)	≥90%	60–89%	<60%
3. Participant retention	% of participants that complete outcome measures at 12 weeks	60 (all recruited patients)	≥90%	60–89%	<60%

### Withdrawals of participants

It will be made clear to all potential participants through the consent form and discussions that they are free to withdraw from study participation at any point, and that they are not required to provide a reason for this.

### Intervention

The intervention is a personalised, holistic follow-up programme of care. It has been developed for a diverse population with differing stroke aetiologies and social contexts and for this reason was guided by the NIHR/MRC framework for complex interventions ^18^ alongside the framework of actions developed by O’Cathain and colleagues ^35^. The behavioural theory chosen to underpin this intervention is self-determination theory (SDT). This theory aims to increase autonomous motivation, which has been shown to be associated with improvements in initiating and sustaining healthy behaviours ^36– 38^. The techniques described by SDT aim to support a person’s three basic psychological needs for competence, autonomy, and relatedness ^39^. It highlights the importance of considering a person’s biological, social, and cultural contexts, which is critical given the diversity of the stroke population. The follow-up appointments will address knowledge gaps, provide support, education, guidance, signposting, and onward referrals as needed. A detailed description as per the TIDieR guidelines for the intervention and control groups is provided in the Open Science Framework (OSF) data repository. https://osf.io/gnw57/

The hypothesized mechanisms for action of the intervention are underpinned by self-determination theory and are:

1) **Enhanced, personalised communication** – this requires listening to and understanding the participants priorities and needs. It requires an understanding of a person’s health literacy and education level. Communication is personalised and adjusted according to the participants needs.

2) **Partnership working** – this approach changes the power dynamic in healthcare interactions and moves away from traditional hierarchical, paternalistic approaches and facilitates the pursuit of personalised goals. It involves linking the participant in with or referring them on to other support services in the community. This approach helps to address adjustment to life after stroke and can mitigate against feelings of isolation and abandonment. It aims to support a sense of belonging, feeling cared for and connected with others.

3) **Enablement** – by focusing on communication and partnership working a participant is supported to have freedom of choice to engage in actions that are congruent with their values and priorities. They are given the tools and knowledge to influence the outcomes that are important to them. This supports intrinsic motivation to initiate and sustain behaviour that supports health and wellbeing.

### Materials and procedures

A training manual for those delivering the intervention has been developed and can be found in the OSF data repository in the intervention delivery folder, accessed here: https://osf.io/gnw57/

The manual describes the principles of SDT and how the techniques can be employed during follow-up appointments. It also describes the key knowledge and skills that are required by the person delivering the intervention and outlines the domains of functioning to be covered in the assessment conducted at the follow-up appointments. Topic areas for discussion in each follow-up appointment are listed, and guidance is provided when onward referrals are needed. Links to relevant informational resources that the participant may require are included in this manual, as well as what to consider if referring to local community stroke services. An intervention delivery checklist has been created to check off all the actions that need to be taken from recruitment through to outcome measurement at 12 weeks. This is also available in the extended data.

The participants GP will be informed of their involvement in the study and which group they have been randomised to. A brief description of the purpose of the study and what it involves, as well as the researcher’s contact details will be included in the letter sent to the GP.

### Intervention provider

For this feasibility study, the lead researcher, who is an experienced stroke occupational therapist, will deliver the intervention. The intention, however, is that this intervention could be delivered by other allied health professionals (AHPs) or nurses with experience working with the stroke population. Therefore, the training manual is designed to address the training needs of both nurses and AHPs. This will be reviewed by nurses and AHPs to obtain feedback on the content, layout, and general utility in supporting delivery of the intervention.

### How, when and where

The mode of delivery of the intervention will focus on inclusivity. To ensure the delivery of a personalised intervention, follow-up appointments will be offered face-to-face in person at the hospital, participant’s home, or a neutral venue in the community, or as a telephone or video call appointment. Participants will have the option of completing their twelve-week outcome measurement in person or alternatively electronic versions of the outcome measures will be sent to participants for their completion. The preferred modality will be noted and will inform a future definitive trial. Where signposting to online information resources or digital Apps is indicated this will form part of the intervention. This will however only be utilized with those that are familiar with and have easy access to these technologies.

The intervention is composed of two follow-up appointments and telephone access to the researcher to address questions that may arise between appointments. The researcher has direct access to the clinical team and stroke clinical specialist nurses if medical concerns are raised. This may help to address feelings of abandonment that are frequently described in the literature. Currently it is very difficult to have ad hoc contact with the stroke clinical team after discharge. Participants in this group will receive referral to the Stroke Association Connect Service and six-month stroke review referrals as part of standard care. The formal follow-up appointments will occur at 2 and 6 weeks after recruitment and will last from 30 to 60 min, depending on the identified needs.

Appointment 1 (two weeks after discharge):

- Understand the participant’s priorities and expectations for the appointment.
- Review functional and psychosocial status. This will provide information on the early impact of stroke, determine what support the participant has in the community, and provide insights into how they are adjusting to their new diagnosis.
- Address the participant’s knowledge gaps regarding stroke, medication, and secondary prevention.
- Agree onward referrals if indicated and action plan for participant and clinician.
- Written summary sent to GP with topics discussed and action plan agreed.
- Summary of discussion points provided to participant when required or requested.
- Complete an early post-stroke quality of life measure (patient-reported outcome measurement information system, PROMIS10)

Appointment 2 (six weeks after discharge):

- Understand the participant’s priorities and expectations for appointment.
- Review actions from first appointment.
- Address any new or persistent information gaps.
- Review functional and psychosocial status, with a focus on potential hidden impairments that may impact return to work, activities of daily living, relationships, or general well-being.
- Agree onward referrals and action plan.
- Summary of discussion points provided to participant when required or requested.
- Written summary and plan to be sent to GP with topics discussed and action plan agreed.

Examples of onward community-based referrals that may be needed include referral to the community stroke therapy team. This may be highlighted when the person is back at home and engaging in their everyday activities and they then become aware of higher-level difficulties that were not detected initially. The hidden impacts of stroke such as fatigue and mood issues are also more likely to become apparent after a period of time at home. The UK and Ireland National Clinical Guidelines for Stroke (2023) now support the need for referral for ongoing rehabilitation at any time point after a stroke if new goals are identified. Other referrals to voluntary sector organisations for information, peer support or exercise groups can also be made if indicated.

***Tailoring***

The content and direction the appointment takes is guided by the manual, but will be tailored by the participant’s own priorities, knowledge gaps, and functional difficulties experienced since their stroke. Information delivery will be tailored to general literacy and health literacy. The participants’ family members or partners will be able to join follow-up appointments if they wish.

***Baseline and outcome measurement***

Baseline measures for both groups will be conducted by the OT in the hospital where possible, but if the person is discharged quickly or seen in the RAC, then they will be conducted at the persons home or in the clinic. Baseline assessment will be conducted within one week of the stroke. The location of the final outcome measurement will be agreed upon by the participant and could be in their home or in the hospital.

Measurement will be taken by the OT delivering the intervention. Participants will be invited to participate in qualitative interviews after the 12-week outcome measurement period.

***Control group***

The participants in the control group will receive standard care. This involves the provision of a printed information sheet on therapy services that exist in their locality and referral to the Stroke Association Connect service, which makes telephone contact with the person after discharge. A six-month stroke review referral is also sent, but currently, this is not consistently provided across all localities. In addition to standard care, those in the control group will be provided with a mobile number and email address for the lead researcher to enable easy contact during the 12-week duration of the study if questions arise relating to their stroke or its effects. If referrals onto community stroke therapy services are indicated at the 12-week outcome measurement appointment then they will be made at that time.

### Primary feasibility outcomes

The time points at which these measurements and secondary feasibility outcomes are taken are presented in the schedule of events table ( Table 5).

**Table 5.** Schedule of events table.

	TIMEPOINTS					
PROCEDURES	T0 0 weeks	T1 2 weeks	T2 4–5 weeks	T3 6 weeks	T4 12 weeks	T5 12–14 weeks
Screening	x					
Consent	x					
Demographics and EDI data	x					
Randomisation	x					
**Intervention**						
Appointment 1		x				
Appointment 2				x		
**Assessment**						
*Primary outcomes:*						
-Recruitment	x					
-Adherence				x		
-Retention					x	
*Secondary outcomes*						
Stroke Knowledge	x				x	
HCCQ	x				x	
PROMIS10		x			x	
PEI SA01					x	
Qualitative interviews						x
**Other variables being measured**						
mRS routinely collected	x				x	
Cognition screen	x				x	
Training material feedback				x		
Mood measures						
PHQ2	x					
PHQ9					x	
GAD7					x	
Economic info						
Health and social care usage		x		x	x	
Work status	x				x	
Admin, programme delivery time	x	x	x	x	x	x
Qualitative interview						x

Table 5 key: HCCQ-Healthcare Climate Questionnaire, PROMIS-10-Patient-reported outcome measurement information system, PEI-Patient enablement instrument, SA01- Substantial amendment 1, mRS-modified Rankin scale, PHQ2-Patient health questionnaire 2, PHQ9-Patient health questionnaire 9, GAD7-Generalised anxiety disorder

1. Recruitment rate (Time point 0 = T0): Proportion of participants randomised relative to the total number meeting the inclusion criteria. Reasons for declining participation will be recorded. The ability to gather baseline and EDI data will be noted as will acceptability of randomisation.

2. Treatment adherence rate (Time point 3 = T3): Proportion of participants in the intervention group who attend both follow-up appointments. When people drop out reasons for this will be obtained.

3. Participant retention rate (T4) - Proportion of all participants that complete the follow-up questionnaires.

### Secondary feasibility outcomes

1. To complete patient reported outcome measures and review their acceptability, accessibility, and responsiveness to inform the primary outcome for a main trial.

i. PROMIS10 - Patient-reported outcome measurement information system. This quality-of-life measure was chosen because it has been found to be more sensitive in people who have mild strokes ^40, 41^ (T1 and T4).
ii. HCCQ - Health Care Climate Questionnaire Short Form – This is a measure of self-determination, particularly autonomy support in health care appointments ^42^ (T0 and T4).
iii. PHQ9 – Patient Health Questionnaire – measure of depression ^41, 43^ (T4).
iv. GAD7 – Generalised Anxiety Disorder – measure of anxiety ^44^ (T4).
v. PEI - Patient Enablement Instrument – measures whether an appointment with a healthcare practitioner impacts a person’s ability to manage their illness ^45^ (T4).

2. To assess stroke knowledge, questions used relate to information that should be provided to patients in their personal stroke record available at: https://www.stroke.org.uk/sites/default/files/professionals/jn_2223-055_-_personal_stroke_record.pdf This document (patient passport) was developed by the Stroke Association in collaboration with stroke survivors, their families, clinicians, and NHS England. The stroke knowledge questions that will be asked are:

i. What type of stroke did you have?
ii. What treatment have you had for your stroke?
iii. Where in your brain did the stroke happen?
iv. How has your stroke affected you?
v. How can you reduce your risk of another stroke?
vi. What new medication are you on?
vii. What are the medications for?
viii. What should you do if you experience symptoms of another stroke?

3. To complete the cognitive screening assessment, the Montreal Cognitive Assessment ^46, 47^ (MoCA) will be conducted in person where possible otherwise over the telephone or video call (T-MOCA ^48^) (T0 and T12).

4. To determine the feasibility of gathering self-reported health and social care usage data (attendance at GP and A&E for stroke-related concerns and new packages of care).

5. To complete qualitative interviews (T5).

6. To determine treatment costs based on appointment, administration and travel time.

7. To note which mode of follow-up is most preferred by participants and whether outcome measures can be effectively completed over the telephone.

8. To obtain feedback on the training manual and intervention delivery checklist from therapy and nursing staff.

### Qualitative data analysis

Participants in the control and intervention groups will be invited to participate in qualitative, semi-structured interviews. The focus of this will be to explore and understand the experience of receiving a diagnosis of stroke and life after stroke for those who received the intervention and those who did not. We will also seek to understand whether the intervention delivered was acceptable and how it could be improved. We will aim to conduct interviews with 15 to 20 participants.

Purposive sampling will be used to ensure that the views of participants from both groups and with a variety of demographic characteristics and experiences after their stroke are obtained. The interviews will be recorded and transcribed verbatim. The interviews will be face-to-face, either on video calls, in the clinic, or at the person’s home. A topic guide will be used, and the interviewer will maintain a field diary to capture reflections and thoughts.

We will review the quantitative measures alongside subjective reports from the participants to evaluate whether there is alignment of the qualitative perspectives and quantitative results. The planned purposive sampling strategy will enable the researcher to interview those who seemed to cope well with the transition back to their lives as well as those that appear to be having more adjustment difficulties. This will capture a diversity of experiences and perspectives.

We will explore whether the themes that arise from the qualitative data are captured in the quantitative measures used. Key concepts and themes that are prominent in the qualitative data will help the researcher to understand what matters to the participants. This also creates an opportunity for participants to introduce topics that may not be reflected in the outcome measures used in the feasibility study. Through these processes we aim to increase the likelihood of identifying a primary outcome for a subsequent definitive trial that is sufficiently nuanced to capture efficacy in a socio-culturally diverse population.

Alongside the study participants, family members that have supported the participant in the research study will be invited to participate in a separate interview. We will aim to conduct at least five interviews with family members.

The data analysis approach will be thematic using the six phases described by Braun and Clarke ^49^. This involves familiarising oneself with the interview transcripts to obtain initial ideas and thoughts and then producing initial codes from the data. Following this, the researcher looks more broadly at the codes to find themes in the data, comparing and contrasting findings across interview transcripts to consider any commonalities, differences, patterns, expected, and unexpected themes. These will be reviewed, using all supporting data to check for credibility and coherence, before refining the final themes and confirming how they interact and relate to each other prior to writing up the findings ^49^. Data will be analysed inductively, but emerging themes will also be considered in relation to Self-Determination Theory to assess the coherence of findings with the theory underpinning the intervention. Computer-assisted qualitative data analysis software (NVivo 14) will be used to support the analysis. Techniques to ensure rigour, such as reflexivity, will be used and reported to understand the researcher’s contextual, personal, and interpersonal influences on the research process ^50^. Peer reviewing of data with a clinical and academic colleague will be conducted, and where perspectives expressed are not clear during data analysis, these will be checked with the participants. Observations and comments noted during the follow-up appointments and the final outcome measure appointment will be referred to during data analysis to add further contextual information to the analysis. 

### Data management

All participants will be assigned a participant ID at the point of consent. Personal data and linking codes will be stored in separate locations using encrypted digital files and password-protected folders. The Case Report Forms (CRF) and questionnaires will only contain participant ID. Paper consent forms, pseudonymised CRFs, and questionnaires will be stored in secure NHS offices in locked cabinets. Scanned copies of the paper consent forms and questionnaires will be stored on secure NHS servers. All data will be collected in accordance with the Data Protection Act 2018 and in line with general data protection regulations (GDPR). Consent will be sought and in place for monitors and auditors from regulatory authorities to have access to identifiable information where relevant. 

Encrypted NHS devices will be used to record the interviews. Transcription of qualitative interviews will involve the transfer of voice recordings to a transcription service that has a data-sharing agreement with the sponsor. The participant ID will be used to label the audio files.

All study documentation will be archived in accordance with the sponsor policy. Data will be destroyed after 5 years.

### Statistical methods

The data obtained will be reported descriptively, and analysis will be conducted using Statistical Package for Social Sciences (SPSS) version 29. The primary analysis will focus on the feasibility outcomes and be presented as proportions with confidence intervals. The monthly recruitment rates will also be noted. Retention data will be noted by group (control and intervention) as well as overall and presented as proportions.

Baseline demographics and clinically relevant data will be reported to enable the understanding of participant characteristics and to enable other services to draw comparisons with their patient demographics.

The responsiveness of the outcome measures to change will be reviewed using confidence intervals for changes from baseline in each group and for between-group comparisons of changes. The numerical results and perceived relevance of the outcome measures used in this feasibility study will be considered. It should be noted that for the current feasibility study the outcome assessor is not blinded, and this has the potential to introduce bias in the assessment. The plan for a future definitive trial would involve a blinded outcome assessor.

The quantitative results will be considered alongside the qualitative findings to illuminate any individual or contextual insights from interviews that help to explain quantitative data. All of these will inform the choice of the primary outcome for a subsequent definitive trial evaluating efficacy.

The economic evaluation and feasibility of self-reported healthcare use will be explored. We will consider the treatment, administration, and clinician travel time to estimate the cost of delivering the intervention. Healthcare usage (GP and A&E attendance for stroke-related concerns) will be obtained from both groups. Self-report data will also capture those that are back at work in their previous capacity, as well as those that have not returned to work or are working in a part-time capacity. We will also capture data where packages of care have been started to support someone at home following their stroke.

### Data monitoring

The study is sponsored by Imperial College Healthcare NHS Trust (ICHNT) and has been reviewed and received approval by the Imperial College London and ICHNT Joint Research Office. The principal investigator will ensure day-to-day management of the trial and will be overseen by the Chief Investigator. The study management group will have an independent chair and two lay members. It will monitor the progress of the trial, recruitment rate, adherence to the protocol, and completion of outcome measures to ensure that the study is progressing as planned. An independent data-monitoring committee is not indicated.

### Audits

The study may be subject to audit by Imperial College Healthcare NHS Trust under their remit as sponsor and other regulatory bodies to ensure adherence to Good Clinical Practice (GCP) and the UK Policy Framework for Health and Social Care Research.

### Dissemination

Dissemination will be targeted and tailored to different audiences. The findings of this feasibility study will be disseminated in a plain English summary to study participants at two time points: at the end of recruitment and after data analysis. Dissemination to other key stakeholders will occur via team updates and presentations of findings in local, regional, and national stroke meetings. Another avenue for dissemination will be through professional networks and social media platforms. The findings of the feasibility study and qualitative interviews will be shared at relevant conferences and in open-access peer-reviewed journals to gain the maximum impact.

### Trial status

Closed to recruitment. Follow-up appointments, outcome measurements, and qualitative interviews are being conducted.

## Ethics and consent

The study received ethical approval from the Hampstead Research Ethics Committee (REC) and the HRA and Health and Care Research Wales on 24/05/2023. REC reference number: 23/LO/0424. The IRAS Project ID is 321584. Documas No: 23CX8211. The NHS confirmation of capacity and capability was received on 21/07/2023.

A substantial request for protocol amendment was submitted on 01/11/2023. This involved adding the Patient Enablement Index to the outcome measures at 12 weeks and including caregivers or family members in the qualitative interviews. If a family member is involved in the recruitment process, baseline measurement, and follow-up appointments, they will be invited to participate in a qualitative interview at the end of the study.

The researcher will go through the participant’s information sheet and address any questions that arise. Time will be allowed if the potential participant wants to talk to the family or needs more time to consider their involvement in the study. Translators will not be used, but for those with limited English proficiency, family members who speak sufficient English will be used to support the consent process.

Feedback from an underserved community highlighted the fear and distrust that existed in these communities when presented with complex participant information sheets. An accessible supplementary information sheet will be used alongside the full participant information sheet and consent form to improve the accessibility of the study information. The consent form, accessible supplementary material, and participant information sheet can be found in the Open Science Framework data repository https://osf.io/gnw57/

## Data Availability

There are currently no data associated with this feasibility study. The reporting guidelines used in this study are the SPIRIT guidelines, and TIDieR checklist. These can be found in the Open Science Framework Data Repository ^51^ https://osf.io/gnw57/ The following extended data are also available in the Open Science Framework data repository: consent form (participant and family member), participant information sheet (for family and participant), accessible information sheet, study protocol, training manual and the assessment and intervention delivery checklist. Data are available under the terms of the Creative Commons Attribution 4.0 International license (CC-BY 4.0).
