# Supplementary material for: Improving follow-up care for people after minor stroke using early personalised care: A protocol for a randomised, mixed-methods, feasibility study
Source: NIHR Open Res. 2025 Feb 26;4:44. Originally published 2024 Aug 1. [Version 3] doi: 10.3310/nihropenres.13649.3 (PMC11868739; doi:10.3310/nihropenres.13649.3)
Supplement: Peer review history

# Reviewer report: Reviewer response for version 3

**DOI:** 10.3310/nihropenres.15143.r34814

**Published:** 2025-02-27

**Authors:** Niamh A. Merriman

**Affiliations:** 1 School of Public Health, Physiotherapy and Sports Science, University College Dublin, Dublin, Ireland

Thank you to the authors for considering my comments and revising the paper. I have no further comments and recommend that the paper is indexed.

Is the study design appropriate for the research question?

Yes

Is the rationale for, and objectives of, the study clearly described?

Yes

Are sufficient details of the methods provided to allow replication by others?

Yes

Are the datasets clearly presented in a useable and accessible format?

Not applicable

Reviewer Expertise:

Complex intervention development; evidence synthesis; cognition; ageing; post-stroke cognitive rehabilitation

I confirm that I have read this submission and believe that I have an appropriate level of expertise to confirm that it is of an acceptable scientific standard.

# Reviewer report: Reviewer response for version 2

**DOI:** 10.3310/nihropenres.15013.r34334

**Published:** 2025-02-10

**Authors:** Niamh A. Merriman

**Affiliations:** 1 School of Public Health, Physiotherapy and Sports Science, University College Dublin, Dublin, Ireland

The authors present a well written and detailed protocol for a randomised, mixed methods, feasibility study of a complex intervention to improve follow-up care for people with minor stroke. This a very important topic and I look forward to seeing the results of feasibility study.

 I would recommend some minor amendments to improve clarity:

**Abstract**

 I would be explicit that the NIHR/MRC framework for complex intervention development and the framework of actions informed the design of the intervention.

**Introduction**

 Under the heading ‘what is a minor stroke?’, I would recommend defining transient ischaemic attack at this stage. It would also be useful to the reader to know what similarities and differences in treatment there are for minor stroke or TIA and stroke as well.

 In this section, the authors state they will recruit patients with an NIHSS score of ≤5 at 24 hours post-admission, however the eligibility criteria specify an NIHSS score of ≤5 at 2 hours post-post. It would be good to clarify the time point and move this information to the methods section.

 Similarly, the authors discuss their aim mid-introduction and that is best placed with the study objectives section.

 I think it would be worthwhile to include a definition of complex interventions.

 I think some more information on what constitutes usual care would be informative to the reader. The authors mention the 6-week review, but it would be good to include the National Clinical Guidelines for Stroke recommendation of 6-month stroke review as well since this is the usual care timeframe mentioned in the methods section.

**Methods**

 It would be useful to the reader to include a table outlining what the different NIHSS scores mean, similar to the table describing the MRS. Also, a key is needed for the abbreviations in each of the tables.

**General comments**

 I wish the authors the best of luck with this very worthwhile project.

Is the study design appropriate for the research question?

Yes

Is the rationale for, and objectives of, the study clearly described?

Yes

Are sufficient details of the methods provided to allow replication by others?

Yes

Are the datasets clearly presented in a useable and accessible format?

Not applicable

Reviewer Expertise:

Complex intervention development; evidence synthesis; cognition; ageing; post-stroke cognitive rehabilitation

I confirm that I have read this submission and believe that I have an appropriate level of expertise to confirm that it is of an acceptable scientific standard, however I have significant reservations, as outlined above.

# Response

**Published:** 2025-02-21

**Authors:** Jennifer Crow

**Affiliations:** Brain Sciences, Imperial College London, London, England, UK

Dear Dr Merriman

 Thank you for the time you have taken to review this paper and for your helpful comments. Please find our response below to all the points you have raised. We present each suggestion you have made or question you have asked and then the relevant current text in the manuscript. We then present the changes we have made based on your suggestions. Text added to the original manuscript is underlined. 

 1.Your suggestion:

 Abstract: I would be explicit that the NIHR/MRC framework for complex intervention development and the framework of actions informed the design of the intervention.

 Current text: This complex intervention is underpinned by self-determination theory, which forms a framework for delivery of the intervention.

***Changes made based on your suggestion:*** *The design of this complex intervention is informed by the NIHR/MRC framework for complex intervention development and the framework of action described by Alicia O’Cathain and colleagues.* *The intervention is underpinned by self-determination theory, which forms a framework for delivery of the intervention.*

 2. Your suggestion:

 Introduction: Under the heading ‘what is a minor stroke?’, I would recommend defining transient ischaemic attack at this stage. It would also be useful to the reader to know what similarities and differences in treatment there are for minor stroke or TIA and stroke as well.

 Current text: In this feasibility study, we will use the tissue-based definition for minor stroke (a stroke visible on imaging), and in terms of impairment scores we will recruit patients with a National Institute of Health Stroke Scale (NIHSS) score ≤ five at 24 hours post admission.

***Changes made based on your suggestions*** *: In this feasibility study, we will use the tissue-based definition for minor stroke (a stroke visible on brain imaging).* *The definition we use for TIA is the presence of transient neurological symptoms which appear to have fully resolved within 24 hours and the absence of tissue damage on brain imaging. The National Institute for Health Stroke Scale (NIHSS) score is an impairment scale which is widely used in clinical practice and research to define minor, moderate or severe stroke (see Table 1)* *(1)* *. The most commonly used cut off points for minor stroke in research studies are NIHSS* *≤* *3 or NIHSS* *≤* *5* *(2)* *.We will be using NIHSS* *≤ 5 as our cut off point in this study.*

*In our service the medical management of those with both minor stroke and TIA focuses on the identification and management of vascular risk factors. Both people with a diagnosis of TIA or minor stroke will have a therapy assessment on the HASU. Only those with a minor stroke are referred for a six-month stroke review. Community stroke therapy teams that subscribe to the time-based definition for TIA will usually not accept referrals for people with a diagnosis of TIA.*

 Ref added: 1. Schwartz JK, Capo-Lugo CE, Akinwuntan AE, Roberts P, Krishnan S, Belagaje SR, et al. Classification of Mild Stroke: A Mapping Review. PM R. 2019;11(9):996-1003.

 Ref added: 2. Leng X, Wang D. Editorial: minor stroke is not minor. Stroke Vasc Neurol. 2023;8(3):175-7.

 3. Your question:

 Introduction: In this section, the authors state they will recruit patients with an NIHSS score of ≤5 at 24 hours post-admission, however the eligibility criteria specify an NIHSS score of ≤5 at 2 hours post-post. It would be good to clarify the time point and move this information to the methods section.

 Our response:

 Thank you for highlighting this inconsistency, we have now corrected this. The correct time point is NIHSS score of ≤5 at 2 hours post admission.

***Changes made based on your suggestion:***

*We have moved this to:*

*Methods:*

*Setting*

*Recruitment:* *Participants with NIHSS score of ≤5 at 2 hours post admission and meeting the other inclusion criteria will be recruited from the HASU and RAC.*

 4. Your suggestion:

 Introduction: The authors discuss their aim mid-introduction and that is best placed with the study objectives section.

 Our response:

 We agree that this should be in the study objectives section and so this has been removed from the introduction and placed in the study objectives section.

 5. Your suggestion:

 Introduction: I think it would be worthwhile to include a definition of complex interventions.

 Our response:

 We agree that including a definition of complex interventions will enhance the manuscript.

 Current text: nothing explained about what a complex intervention is in the original text.

**Changes made based on your suggestion:**

 We have added a short paragraph in the introduction section between the paragraph ‘What is a minor stroke?’ and the paragraph ‘What happens in current practice?’  which we have headed with ‘What is a complex intervention?’

*What is a complex intervention?*

*Many interventions used in healthcare are considered to be complex interventions, whereby the intervention consists of a number of different interacting components, each targeting different areas of behavioural change or aspects of health and wellbeing. Complex interventions allow a degree of flexibility as they are applied in different contexts or different systems to a wide range of individuals with varying levels of health literacy and from a diverse range of communities (* *3)* *.*

 Ref added: 3. Skivington K, Matthews L, Simpson SA, Craig P, Baird J, Blazeby JM, et al. A new framework for developing and evaluating complex interventions: update of Medical Research Council guidance. BMJ. 2021;374: n2061

 6. Your suggestion:

 Introduction: I think some more information on what constitutes usual care would be informative to the reader. The authors mention the 6-week review, but it would be good to include the National Clinical Guidelines for Stroke recommendation of 6-month stroke review as well since this is the usual care timeframe mentioned in the methods section.

 Our response: We have added information on the 6-month stroke review which is offered as part of usual care in the methods section.

 Current text: Current medical follow-up appointments are brief and focus specifically on medical issues and outstanding medical investigations. Ideally, these should occur 6 weeks post-discharge, but in practice, the wait for these appointments is often longer. These appointments rarely have multidisciplinary input; therefore, other issues relating to hidden effects, adjustment to life after stroke, and secondary prevention are seldom addressed.

***Changes made based on your suggestion:*** *National Clinical Guidelines for Stroke (2023) recommend that all people after stroke should receive a holistic and structured 6-month review (* *4)* *.  People with a diagnosis of TIA are seldom offered this review and it can be more challenging for these individuals to access community stroke services.*

 Ref added: 4. Intercollegiate Stroke Working Party. National Clinical Guideline for Stroke for the UK and Ireland London2023 [Available from: https://www.strokeguideline.org/app/uploads/2023/04/National-Clinical-Guideline-for-Stroke-2023.pdf

 7. Your suggestion:

 Methods: It would be useful to the reader to include a table outlining what the different NIHSS scores mean, similar to the table describing the MRS. Also, a key is needed for the abbreviations in each of the tables.

 Our response: We have now added Table 1 with the NIHSS scores as suggested. We have also included abbreviations in a key for each of the tables.

**Changes made based on your suggestions:**

 The information below is presented in a table in the manuscript.

*Table 1: Stroke severity - National Institute of Health Stroke Scale Score*

*0 = No Stroke symptoms*

*0-4 = Minor Stroke*

*5-15 = Moderate Stroke*

*16-20 = Moderate to Severe Stroke*

*21-42 = Severe stroke*

 Ref added: 5. Zhuo Y, Qu Y, Wu J, Huang X, Yuan W, Lee J, et al. Estimation of stroke severity with National Institutes of Health Stroke Scale grading and retinal features: A cross-sectional study. Medicine (Baltimore). 2021;100(31):e26846.

*Table 2. Participant data collection domains*

*Key: mRS-modified Rankin Scale, PHQ2-patient health questionnaire 2, EDI-ethnicity, diversity and inclusion, NIHSS-National Institute of Health Stroke Scale, MoCA-Montreal cognitive assessment.*

*Table 4: Schedule of Events Table*

*Key: HCCQ-Healthcare Climate Questionnaire, PROMIS-10-Patient-reported outcome measurement information system, PEI-Patient enablement instrument, SA01- Substantial amendment 1, mRS-modified Rankin scale, PHQ2-Patient health questionnaire 2, PHQ9-Patient health questionnaire 9, GAD7-Generalised anxiety disorder*

# Reviewer report: Reviewer response for version 2

**DOI:** 10.3310/nihropenres.15013.r33808

**Published:** 2024-12-17

**Authors:** Mathew Reeves

**Affiliations:** 1 Epidemiology, Michigan State University, East Lansing, Michigan, USA

I have read the response from the authors and was impressed with the number of substantive changes made to the protocol in response to my prior comments.  I now approve the final version of this protocol and wish the authors the best of luck for the successful implementation of this important project.

Is the study design appropriate for the research question?

Yes

Is the rationale for, and objectives of, the study clearly described?

Yes

Are sufficient details of the methods provided to allow replication by others?

Yes

Are the datasets clearly presented in a useable and accessible format?

Not applicable

Reviewer Expertise:

NA

I confirm that I have read this submission and believe that I have an appropriate level of expertise to confirm that it is of an acceptable scientific standard.

# Reviewer report: Reviewer response for version 1

**DOI:** 10.3310/nihropenres.14819.r32856

**Published:** 2024-10-10

**Authors:** Mathew Reeves

**Affiliations:** 1 Epidemiology, Michigan State University, East Lansing, Michigan, USA

This is a well written and formatted protocol for a pilot feasibility study of a post discharge intervention trial to improve recovery in mild stroke patients. The justification for the study was well described with sufficient background materials. A scoping review was provided that highlighted the deficiencies of the current published research studies (n= 25) which show an incredible level of heterogeneity in terms of goals, study designs, interventions, outcomes, and results. One thing that could have been given more attention is that few if any interventions to date have shown meaningful improvements in patient outcomes in this population.

 Other specific critiques include:

 Two touch points at 2 and 6 weeks is quite limited.  Opportunities to continue the dialogue and delivery of services across the 12-week period are probably needed.   There appears to be limited use of mHealth technologies to deliver or supplement the intervention, or to collect data.

 It would be helpful if specific examples of community-based referrals that are available to be accessed within the time period of the intervention were identified.

 It is unclear if and how GPs are involved in the study? What information will they receive on both the intervention and control subjects?

 There is also some concern that retention rates will be substantially lower for the control group given that they receive little (or no?) direct contact after enrollment. Investigators should consider adding some minimal ongoing level of engagement with the control group between 2 and 6 weeks.   

 Clarify if the Stroke Association Connect program will be given to both arms? And whether the program itself has been made aware of this trial and the subject’s enrollment?

 I would suggest that a patient-reported measure of physical function be collected at baseline, 2, 6 and 12 weeks, and also that all baseline data collection is standardized to occur at the same time point (preferably soon after returning home).

 It is unclear why the outcome assessment is not being done blinded? Explain and justify. What mitigation steps can be taken to reduce bias?

 Eligibility criteria does not include a baseline NIHSS score?  Why is this?  What is the maximum time allowed between stroke onset and enrollment?

 What is the expected total annual stroke case load at the RAC and HASC?

 Table 3: The >90% recruitment threshold appears to be quite ambitious and may be difficult to reach.

 Table 4. Add fatigue?

 The success or failure of studies like this largely depends on how treatment efficacy is measured. Decision on how a primary outcome measure will be selected are far from easy. More details of how qualitative interviews and data responsiveness will be combined to make the choice is required. What are the hypothesized mechanisms where 2 visits focused on unmet needs can really make a difference?

Is the study design appropriate for the research question?

Yes

Is the rationale for, and objectives of, the study clearly described?

Yes

Are sufficient details of the methods provided to allow replication by others?

Yes

Are the datasets clearly presented in a useable and accessible format?

Not applicable

Reviewer Expertise:

Stroke epidemiologist and trialist with experience with stroke transitions.

I confirm that I have read this submission and believe that I have an appropriate level of expertise to confirm that it is of an acceptable scientific standard, however I have significant reservations, as outlined above.

# Response

**Published:** 2024-11-07

**Authors:** Jennifer Crow

**Affiliations:** Brain Sciences, Imperial College London, London, England, UK

Dear Professor Reeves

 Thank you for taking the time to review our feasibility study protocol and for the helpful feedback and suggestions you have provided. We have responded to all the points you have raised and have made adjustments to our protocol manuscript accordingly to improve the quality, clarity and robustness of the feasibility study being conducted.  See below our point-by-point responses to your comments with our responses labelled as 'response' and manuscript adjustments underlined within the original text, both in italics.

 1.One thing that could have been given more attention is that few if any interventions to date have shown meaningful improvements in patient outcomes in this population.

 RESPONSE: Thank you, this is a very important question to address. We have now added an explanation to the manuscript text to provide more clarity on how the scoping review helped us to identify potential key elements for inclusion in our intervention. Specifically, we describe two studies that were able to demonstrate meaningful improvement in HRQOL at 12 months after stroke.  In our intervention the key elements that we have included are a multidisciplinary focus, personalised care and self-determination theory.

*We conducted a scoping review to identify follow-up programmes, pathways, and services that have been developed for people after minor stroke22. Twenty-five studies were included in the analysis. Interventions often focused on education, secondary prevention, and exercise after stroke, whereas the management of hidden impairments and adjustment to life after stroke were less likely to be addressed. These complex interventions were often poorly described, and descriptions of family involvement, liaison between primary and secondary care, and underpinning psychological or behavioural theories were lacking. The review revealed that it is still unknown how best to organise services to offer personalised timely support and education to people in the community. There were two RCTs included in the scoping review (STROKECARD* *(1)* *and the Taking Charge after Stroke (TACAS) study* *(2)* *) that were able to demonstrate improvements in health-related quality of life at 12 months after the stroke. STROKECARD involved personalised, comprehensive multidisciplinary follow-up at 3 months post stroke and the TACAS follow-up intervention was underpinned by self-determination theory and involved either one or two follow-up appointments. TACAS’s person centred intervention focused on supporting a person to take charge of their recovery through supporting self-empowerment. The key elements from these two studies; multidisciplinary focus, personalised care and self-determination theory are included in the intervention described in this protocol. We believe that this may increase the likelihood of being able to show meaningful improvements in this diverse patient population.*

 2. Two touch points at 2 and 6 weeks is quite limited.  Opportunities to continue the dialogue and delivery of services across the 12-week period are probably needed.   

 RESPONSE: Thank you for this point. The two follow-up appointments are supplemented by direct mobile phone access to the researcher which enables participants to discuss any questions or concerns that arise during the 12-week duration of the study. The researcher in turn has direct access to the clinical team, including the stroke clinical specialist nurses if medical concerns are raised. This is novel because currently it is very difficult to have any ad hoc contact with the stroke clinical team after discharge. Contact is restricted to the follow-up medical appointment which could be 12 weeks after discharge. Due to substantial pressures within primary care, GPs are currently very difficult to access directly, and they often direct questions back to the specialist stroke service. I have provided further clarification of this in the manuscript text as below.

*The intervention is composed of two follow-up appointments and telephone access to the researcher to address questions that may arise between appointments. The researcher has direct access to the clinical team and stroke clinical specialist nurses if medical concerns are raised. This may help to address feelings of abandonment that are frequently described in the literature. Currently it is very difficult to have ad hoc contact with the stroke clinical team after discharge. The fomal follow-up appointments will occur at 2 and 6 weeks after recruitment and will last from 30 to 60 min, depending on the identified needs.*

 3. There appears to be limited use of mHealth technologies to deliver or supplement the intervention, or to collect data.

 RESPONSE: Thank you for raising this important question. We recognise the increasing relevance of these approaches, but in order to be inclusive and avoid digital exclusion we have limited the essential use of mHealth technologies in the intervention delivery and outcome measurement. The option does exist for the follow-up appointments to be conducted virtually as well as the outcome measurement at 12 weeks. In these cases, the participants will be sent electronic versions of the patient reported outcome measures. We will not be excluding the use of online resources and digital Apps but will only signpost participants to them when they are familiar with, and have easy access to, these technologies. In this way we hope to provide accessible and personally tailored follow-up. Below we have tried to make this clearer in the manuscript.

*To ensure the delivery of a personalised intervention* *, follow-up appointments will be offered face-to-face in person at the hospital, participant’s home, or a neutral venue in the community, or as a telephone or video call/virtual appointment. Participants will have the option of completing their twelve-week outcome measurement in person or alternatively electronic versions of the outcome measures will be sent to participants for their completion. The preferred modality will be noted and will inform a future definitive trial. Where signposting to online information resources or digital Apps is indicated this will form part of the intervention. This will however only be utilized with those that are familiar with and have easy access to these technologies.*

 4. It would be helpful if specific examples of community-based referrals that are available to be accessed within the time period of the intervention were identified.

 RESPONSE: We have now clarified this in the manuscript text as below, in the ‘How, when and where’ section of the text which describes what is covered in the follow-up appointments. A GP could potentially also make these referrals but given that a GP appointment in the UK is 9 minutes long it is unlikely that they will have the opportunity to gauge what a person’s post-stroke functional, cognitive or psychological problems might be and they will therefore be unlikely to initiate referrals for ongoing community stroke therapy.

*Examples of onward community-based referrals that may be indicated include referral to the community stroke therapy team. This may be indicated when the person is back at home and engaging in their everyday activities and then becomes aware of higher-level difficulties that were not detected initially. The hidden impacts of stroke such as fatigue and mood issues are also more likely to become apparent after a period of time at home. Within the UK and Ireland the National Clinical Guidelines for Stroke (2023) now support the need for referral for ongoing rehabilitation at any time point after a stroke if new goals are identified. Other referrals to voluntary sector organisations for information, peer support or exercise groups can also be made if indicated.*

 5. It is unclear if and how GPs are involved in the study? What information will they receive on both the intervention and control subjects?

 RESPONSE: The GPs will be informed via letter if one of their patients is involved in this study. They will be informed if the person is in the intervention or control group and will be provided with information on the purpose of the study and what it involves. Contact details of the researcher will also be available on the letter. Following the 1 ^st^ and 2 ^nd^ follow-up appointments the GP will receive a summary letter with the topics discussed and action plan agreed.

*To be included in the materials and procedures section:*

*The participant's GP will be informed of their involvement in the study and which group they have been randomised to. A brief description of the purpose of the study and what it involves, as well as the research team’s contact details, will be included in the letter sent to the GP.*

*Appointment 1:*

*Written summary sent to GP with topics discussed and action plan agreed.*

*Appointment 2:*

*Written summary and plan to be sent to GP with topics discussed and action plan agreed.*

 6. There is also some concern that retention rates will be substantially lower for the control group given that they receive little (or no?) direct contact after enrollment. Investigators should consider adding some minimal ongoing level of engagement with the control group between 2 and 6 weeks. 

 RESPONSE: The ongoing engagement with the control group will be the provision of the mobile number and email address of the researcher. Participants will be informed that they can contact the researcher at any time during the 12-week duration of the study if they have questions arising related to their stroke or its effects. Under normal circumstances it is not possible to have contact with the acute team once a person has left the hospital. All concerns need to be directed to the GP, but GPs may not have received the hospital discharge summary and because the IT systems are not linked they are unable to see what upcoming investigations and appointments have been booked. Having direct access to a professional based in the acute setting is therefore advantageous and offers the opportunity for an ongoing level of engagement. Adjustments made in response to question 2 also provide information on how this will provide an ongoing level of engagement.

*Control group*

*The participants in the control group will receive standard care. This involves the provision of a printed information sheet on therapy services that exist in their locality and referral to the Stroke Association Connect service, which makes telephone contact with the person after discharge. A six-month stroke review referral is also sent, but currently, this is not consistently provided across all localities. In addition to standard care, those in the control group will be provided with a mobile telephone number and email address for the lead researcher to enable easy contact during the 12-week duration of the study if questions arise relating to their stroke or its effects. If referrals onto community stroke therapy services are indicated at the 12-week outcome measurement appointment, then they will be made at that time.*

 7. Clarify if the Stroke Association Connect program will be given to both arms? And whether the program itself has been made aware of this trial and the subject’s enrolment?

 RESPONSE: Both arms are referred to the Stroke Association Connect programme. This programme involves a follow-up telephone call to the person after stroke between 2-4 weeks after the event. The Stroke Association connector provides reassurance, guidance with any immediate concerns, and connects people to ongoing peer support. The connector is not medically trained, and they are not provided with any of the persons medical details except that they have had a stroke. If any medical questions arise the person is directed to speak to their GP. This is part of usual care, and the connector will not routinely be informed of the participants' involvement in the study.

*The intervention is composed of two follow-up appointments and telephone access to the researcher to address questions that may arise between appointments.* *Participants in this group will receive referral to the Stroke Association Connect Service and six-month stroke review referrals as part of standard care.* *Follow-up appointments will occur at 2 and 6 weeks after recruitment and will last from 30 to 60 min, depending on the need*.

 8. I would suggest that a patient-reported measure of physical function be collected at baseline, 2, 6 and 12 weeks, and also that all baseline data collection is standardized to occur at the same time point (preferably soon after returning home).

 RESPONSE: Thank you for this suggestion. We are using the PROMIS10 Global Health patient-reported measure, and this will give a physical health and mental health score at 2 weeks and 12 weeks. This measure is not being used at baseline because participants will largely be recruited within one or two days of their stroke and therefore may not yet have a sense of how their physical function and mental health has been impacted. The modified Rankin Scale is being gathered to give an indication of pre-stroke physical function. As you have highlighted it is important to standardize the timepoint at which the baseline data is collected, we have now included this in the manuscript text. As this is a feasibility study, we are providing a 1 week window from diagnosis to baseline assessment (either in hospital or a few days after getting home). This will enable us to understand participant preference and through qualitative interviews explore the participants' perspective on having the baseline assessments at home versus when still in hospital.

*Outcome measurement: Baseline measures for both groups will be conducted by the OT researcher in the hospital where possible, but if the person is discharged quickly or seen in the RAC, then they will be conducted at the persons home or in the clinic. Baseline assessment will be conducted within 1 week of the stroke. The location of the final outcome measurement will be agreed upon with the participant and could be in their home or in the hospital.*

 9. It is unclear why the outcome assessment is not being done blinded? Explain and justify. What mitigation steps can be taken to reduce bias?

 RESPONSE: Thank you, this is an important point. As this feasibility study is part of a PhD, there is only funding available for one researcher and therefore all measurement and delivery of the intervention is being conducted by the same person. If the results suggest that a future funded definitive trial is possible then outcome assessment will be blinded as there will be a team involved in delivering the study. In this feasibility study the focus is on the feasibility objectives of recruitment, adherence and retention rather than any efficacy measures. However, in acknowledgement of the reviewer's point we have now included in the manuscript an acknowledgement that an unblinded outcome assessor can introduce bias.

*The responsiveness of the outcome measures to change will be reviewed using confidence intervals for changes from baseline in each group and for between-group comparisons of changes. The numerical results and perceived relevance of the outcome measures used in this feasibility study will be considered. It should be noted that for the current feasibility study the outcome assessor is not blinded, and this has the potential to introduce bias in the assessment. The plan for a future definitive trial would involve a blinded outcome assessor.*

 10. Eligibility criteria does not include a baseline NIHSS score?  Why is this?  What is the maximum time allowed between stroke onset and enrolment?

 RESPONSE: Thank you for highlighting this and this is an omission on our part. We have now added this to the inclusion criteria:  NIHSS ≤ 5 at 2 hours post admission to allow those that have had hyperacute interventions and done well to be included in the study. We have also now clarified in the inclusion criteria the maximum time allowed between stroke onset and enrolment.

*Eligibility criteria Inclusion criteria*

*• Adult ≥18*

*• Clinical or radiological diagnosis of minor stroke from a stroke consultant.*

*• Admitted to HASU or seen in RAC.*

*• Pre-admission modified Rankin Score (mRS) of 0-3 (see table below), with no formal package of care prior to stroke. Table 1. Modified Rankin Scale scores*

-
*NIHSS ≤ 5 at 2 hours post admission*

-
*Within one week of receiving stroke diagnosis*

*• Discharge mRS of 0-3.*

*• No onward referral to community therapy team or for package of care.*

*• Has capacity to consent.*

 11.What is the expected total annual stroke case load at the RAC and HASU?

 RESPONSE: Our hyperacute stroke unit (HASU) has approximately 1700 admissions per year, about 1400 of which end up with a diagnosis of stroke. The Rapid Assessment Clinic receives about 160 – 175 referrals every month, of which 50 – 60 are rejected. Many of the referrals are stroke mimics, some are TIA and about 5 per month receive a diagnosis of minor stroke. 

 12. Table 3: The >90% recruitment threshold appears to be quite ambitious and may be difficult to reach.

 RESPONSE: Thank you for this comment and you are correct that it is an ambitious target. We believe that this target for this feasibility study may be achievable for the following reasons:

 - It is only conducted at a single centre and the researcher has good working relationships with both the stroke medical and therapy teams.

 - We have done extensive outreach work with diverse community groups which has improved our understanding of culturally appropriate ways to approach patients from different communities. We believe that this may result in more successful recruitment of participants that reflect the stroke demographic.

 - The personalised nature of the intervention and flexible follow-up appointment arrangements were designed to cause minimum disruption to a person’s daily life, caring responsibilities and work routines.

*The targets may seem ambitious, but due to the limited number of appointments and personalised nature of the intervention, where participants do not need to attend appointments at the hospital, we believe these are achievable. We have carried out extensive work with diverse community groups that has improved our understanding of culturally appropriate ways to approach patients from different communities. Further, this is also a single-centre study, with the lead researcher having established working relationships with the other members of the stroke team.*

 13. Table 4. Add fatigue?

 RESPONSE: As you highlight through this suggestion, fatigue is a commonly reported problem after minor stroke. In order to keep the burden of outcome measure completion as low as possible for the feasibility study we decided that rather than include an additional separate fatigue outcome measure, we would utilise the fatigue question included in the PROMIS-10 v1.2 ('In the past 7 days how would you rate your fatigue on average?') In any future definitive trial, we would include a measure of fatigue that provides more granularity rather than solely determining whether it is present.

 14. The success or failure of studies like this largely depends on how treatment efficacy is measured. Decision on how a primary outcome measure will be selected are far from easy. More details of how qualitative interviews and data responsiveness will be combined to make the choice is required.

 RESPONSE: This will be one of the most important and challenging questions to answer when moving from the current feasibility study to a definitive trial. Thus, a stated secondary objective in this feasibility study is to determine what outcome measures should be used for a future efficacy trial. We will be looking at a number of factors:

- Accessibility: are participants able to understand and complete the outcome measures without support? When extra support is required does this relate to language or understanding of concepts or something different to these.
- Responsiveness to change: we will review the quantitative outcome measures to understand if they reflect any change within and between groups over the duration of the study.
- Qualitative interviews: we will look at how the scores on patient reported outcome measures align with what people report in the qualitative interviews. We will need to explore whether the themes that arise from the qualitative data are captured in the quantitative measures used.
- Below we provide a more detailed description of how we propose to select a primary outcome measure.

*Qualitative data analysis*

*Participants in the control and intervention groups will be invited to participate in qualitative, semi-structured interviews. The focus of this will be to explore and understand the experience of receiving a diagnosis of stroke and life after stroke for those who received the intervention and those who did not. We will seek to understand whether the intervention delivered was acceptable and how it could be improved.*

*Alongside this we will review the quantitative measures alongside subjective reports from participants to evaluate whether there is alignment of the qualitative perspectives and quantitative results. The planned purposive sampling strategy will enable the researcher to interview those who seemed to cope well with the transition back to their lives as well as those that appear to be having more adjustment difficulties. This will capture a diversity of experiences and perspectives.*

*We will explore whether the themes that arise from the qualitative data are captured in the quantitative measures used. Key concepts and themes that are prominent in the qualitative data will help the researcher to understand what matters to the participants. This also creates an opportunity for participants to introduce topics that may not be reflected in the outcome measures used in the feasibility study. Through these processes we aim to increase the likelihood of identifying a primary outcome for a subsequent definitive trial that is sufficiently nuanced to capture efficacy in a socio-culturally diverse population.*

 15. What are the hypothesized mechanisms where 2 visits focused on unmet needs can really make a difference?

 RESPONSE: We agree with the reviewer that the manuscript would be improved with more extensive mechanistic underpinning of the detailed intervention. Below we describe what the proposed mechanisms of action are that underpin how two follow-up appointments might achieve improved knowledge, health and wellbeing. We will add this text to the manuscript after the descriptions of the intervention and tailoring.

*The hypothesized mechanisms for action are underpinned by self-determination theory* *(3)* *. This theory posits that we all have three basic psychological needs for competence, autonomy and relatedness and when these are supported a person’s motivation and engagement are improved.*

*The proposed mechanisms of action within the intervention are:*

*1) **Enhanced, personalised communication** – this requires listening to and understanding the participants priorities and needs. It requires an understanding of a person’s health literacy and education level. Communication is personalised and adjusted according to the individual’s needs, acknowledging that one approach would not meet everyone’s needs.*

*2) **Partnership working** – this approach changes the power dynamic in healthcare interactions and moves away from traditional hierarchical, paternalistic approaches and facilitates the pursuit of personalised goals. It involves linking the participant in with, or referring them on to, other support services in the community. This approach helps to address adjustment to life after stroke and can mitigate against feelings of isolation and abandonment. It aims to support a sense of belonging, feeling cared for and connected with others.*

*3) **Enablement** – by focusing on communication and partnership-working a participant is supported to have freedom of choice to engage in actions that are congruent with their values and priorities. They are given the tools and knowledge to influence the outcomes that are important to them. This supports intrinsic motivation to initiate and sustain behaviour that supports health and wellbeing.*

 1.           Willeit P, Toell T, Boehme C, Krebs S, Mayer L, Lang C, et al. STROKE-CARD care to prevent cardiovascular events and improve quality of life after acute ischaemic stroke or TIA: A randomised clinical trial. EClinicalMedicine. 2020;25:100476.

 2.           Fu V, Weatherall M, McPherson K, Taylor W, McRae A, Thomson T, et al. Taking Charge after Stroke: A randomized controlled trial of a person-centered, self-directed rehabilitation intervention. Int J Stroke. 2020:1747493020915144.

 3.           Ryan R DE. Self-Determination Theory and the Facilitation of Intrinsic Motivation, Social Development, and Well-Being. Am Psychol. 2000;55(1):68-78.
